# Sociodemographic differences in medication use, health-care contacts and sickness absence among individuals with medication-overuse headache

**DOI:** 10.1007/s10194-012-0432-y

**Published:** 2012-03-17

**Authors:** Pernilla Jonsson, Mattias Linde, Gunnel Hensing, Tove Hedenrud

**Affiliations:** 1 Institute of Medicine, Sahlgrenska Academy, University of Gothenburg, P. O. Box 453, 405 30 Gothenburg, Sweden; 2 Department of Neuroscience, Norwegian University of Science and Technology, Trondheim, Norway; 3 Norwegian National Headache Centre, St. Olav’s University Hospital, Trondheim, Norway; 4 Institute of Neuroscience and Physiology, Sahlgrenska Academy, University of Gothenburg, Gothenburg, Sweden

**Keywords:** Headache, Medication-overuse headache, Epidemiology, Educational status, Medication use, Health-care contacts

## Abstract

The objective of this study was to analyse sociodemographic differences in medication use, health-care contacts and sickness absence among individuals with medication-overuse headache (MOH). A cross-sectional, population survey was conducted, in which 44,300 Swedes (≥15 years old) were interviewed over telephone. In total, 799 individuals had MOH. Of these, 47 % (*n* = 370) only used over-the-counter medications. During the last year, 46 % (*n* = 343) had made a headache-related visit to their physician and 14 % (*n* = 102) had visited a neurologist. Among individuals aged <30 years, the number of days/month with headache was greater than the number of days with medication use, whereas the opposite was true for those ≥30 years. Both the proportion using prophylactic medication and the proportion having consulted a neurologist were smaller among those who only had elementary school education than among those with higher education (*p* = 0.021 and *p* = 0.046). Those with a lower level of education also had a higher number of days/month with headache and with medication use than those with a higher educational level (*p* = 0.011 and *p* = 0.018). The MOH-sufferers have limited contacts with health-care and preventive measures thus need to include other actors as well. Particular efforts should be directed towards those with low educational levels, and more research on medication use in relation to age is required.

## Introduction

Paradoxically, medications that normally relieve headache may also increase the frequency of headache if overused, causing so-called medication-overuse headache (MOH) [1]. Medication-overuse headache develops in individuals with primary headache disorders who overuse acute headache medication [2]. The MOH-sufferers have a headache at least 15 days/month and the disorder has a considerable impact on their quality of life [2–5].

In order to manage their situation, individuals with MOH use large quantities of medications, by definition triptans, ergots, opioids, or combination analgesics on at least 10 days/month or simple analgesics at least 15 days/month [2]. Addictive behaviour has been discussed in relation to MOH, particularly among those overusing psychotropic substances, and it has been suggested that such users should be regarded as a specific, more severe subgroup of MOH [6–8]. The MOH is one of the forms of headache that most frequently causes patients to seek care at headache centres [9–11]. Around 30 % of patients seen at headache centres have MOH [11]. However, some headache sufferers never seek medical care and many of those who do, do not return for follow-up visits [12, 13]. The MOH-sufferers also report more sickness absence and more days with reduced productivity at work than migraineurs [14].

In previous studies on MOH, little attention has been paid to sociodemographic differences. The prevalence of the disorder is 1–2 % in the general population and it is known that it is more prevalent among women than men, that there are age-differences in prevalence, and that it is more common among those with a low socioeconomic status [4, 15–17]. However, no studies have looked more closely at these differences in relation to medication use, health-care contacts and sickness absence. Such knowledge is important in order to shed light on how resource use is distributed and to identify groups for possible future interventions. In addition many studies, particularly those regarding health-care contacts [9–11], are based on clinical samples, and there is thus a need for population-based studies in this area. The aim of this study was therefore to analyse sociodemographic differences in medication use, health-care contacts and sickness absence among individuals with MOH. These factors were analysed from a population perspective in Sweden.

## Methods

### Sampling and interview

Data were collected through a national telephonic survey conducted by Sifo Research International, a Swedish opinion poll agency. This survey has an omnibus design. It runs continuously, reaches approximately 1,000 individuals per week and provides a means for data collection for different research projects, companies, and organisations. Sampling for this study was performed between March 2009 and March 2010 and consisted of randomised sampling in two steps. In the first step, a household was selected and in the second step, a household member from that specific household was singled out. The basis for selection was the current national telephone directory. Households without telephones were not included. A computer programme randomly chose numbers in the telephone directory. It also constructed new telephone numbers by adding digits to those already chosen. This procedure ensured inclusion of numbers that were not listed in the directory. If the number led to a company or a public authority, or if there was an unobtainable tone, a new number was chosen. Numbers with no reply were called again later and if there was still no reply, they were replaced by new numbers. When the interviewer came into contact with a household, he or she initially collected information on the number of Swedish-speaking household members aged ≥15 years, and the computer programme randomly chose one of these individuals for the interview.

A large group of lay interviewers aged ≥18 years, with an average of 2 years of interviewing experience, administered the questionnaire. They introduced the interview by explaining that it was a survey by Sifo, covering several different areas, which would last approximately 5–25 min. Verbal informed consent was obtained and all had the right to decline participation or to refuse to answer specific questions without explanation. The study protocol was approved by the Regional Ethical Review Board in Gothenburg.

### Questionnaire

All respondents were asked background questions concerning sex, age, and the highest level of education (elementary school, high school or university). The interviewers introduced the part of the survey related to this study by explaining that the questions concerned headache and came from the University of Gothenburg. This part of the survey began with two screening questions and only respondents who passed these were asked further questions. In order to pass, the respondent would have to report headache present on ≥15 days/month and medication use for ≥10 days/month during the past 3 months.

The subsequent interview comprised questions about medication use, health-care contacts, headache-related sickness absence and primary headache.

*Medication use*: The respondents were first asked to name the medication that they most frequently used in order to treat their headache (primary acute medication). They were then asked a series of follow-up questions regarding this medication: frequency of use, form of dosage and whether they bought it on prescription, as over-the-counter (OTC)-medication or both (this variable was dichotomised into “always OTC” and “sometimes or always on prescription”). For medications other than the primary one, only the name and the frequency of use were asked for. The medications reported were divided into five different groups corresponding to the diagnostic criteria of MOH [2]. Addictive behaviour has been discussed in relation to MOH [6–8]. Therefore, in some analyses all medications containing psychotropic substances (alone or in combination with other active compounds) were analysed as one group. There was also a question regarding the use of prophylactic medication.

*Health-care contacts*: The respondents were asked how many times they had visited a physician due to headache during the last year. They were also asked what type of physician they had visited (neurologist or other), number of prescribing physicians and whether any physician had ever informed them that excessive use of acute headache medication could lead to an increased frequency of headache.

*Sickness absence*: Sickness absence was reported as mean number of days/month and person during the last 3 months and only analysed among those aged 18–65 years.

*Headache diagnoses*: The 2006 International Headache Society appendix criteria were used to diagnose MOH and the primary headaches were diagnosed as “migraine” or “other headaches” according to the International Classification of Headache Disorders second edition (ICHD-II) [2, 18].

### Statistical analysis

The IBM SPSS Statistics version 19.0 for Windows was used for all statistical analyses. Differences between percentages were analysed using the Pearson Chi square test. All percentages are valid percentages, i.e. calculated after the exclusion of missing values. Means are presented with standard deviation (SD). Differences between means were tested using the independent sample *t* test. When three or more means were compared, univariate analysis of variance (ANOVA) was used. When a difference was detected, the most appropriate post hoc range test was performed to determine which scores differed. Associations were investigated using univariate logistic regression when the dependent variable was dichotomous and Pearson correlation for continuous variables. The significance level was set to *p* < 0.05.

## Results

### The sample

In total, 44,300 individuals (24,195 women and 20,105 men) were interviewed. Sampling was performed by the method of substitution (the interviewer called a new number if there was no reply) and the total number of telephone calls was not documented. The drop-out rate, defined as individuals who agreed to answer the overall interview but who declined to answer the section regarding headache and medication use, was 1.6 % (*n* = 700). A total of 799 individuals with MOH were identified. Of these, 76 % (*n* = 609) were women and the mean age was 51 years (SD ± 15). The demographic characteristics of the sample are presented in Table 1.

**Table 1:** Population characteristics among 799 individuals with medication-overuse headache (MOH)

	Men (*n*)	Women (*n*)	Total	
			*n*	%
Total	190	609	799	100
Age (years)				
15–20	2	21	23	2.9
21–29	5	41	46	5.8
30–39	22	92	114	14.3
40–49	44	148	192	24.0
50–64	72	212	284	35.5
65–74	27	62	89	11.1
≥75	17	33	50	6.3
Missing	1	0	1	–
Educational level				
Elementary school	86	210	296	37.3
High school	70	245	315	39.7
University	32	150	182	23.0
Missing	2	4	6	–
Primary headache				
Migraine	78	316	394	58.5
Other headaches	76	204	280	41.5
Missing	36	89	125	–

The mean frequency of headache was 22.8 days/month, and 35 % (*n* = 276) reported having a headache every day. Men reported a higher frequency than women (23.7 vs. 22.6 days/month, *p* = 0.033) and those who had only had elementary school education reported a higher frequency (23.8 days/month) than those who had attended high school (22.4 days/month, *p* = 0.011) or university (21.9 days/month, *p* = 0.0021).

### Medication use

**Fig. 1:**
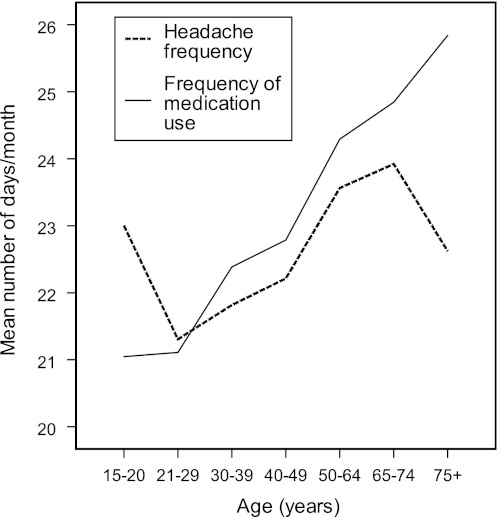
Frequency of headache and of medication use in relation to age, among 799 individuals with medication-overuse headache (MOH). The frequencies are reported as the mean number of days/month over the last 3 months

Daily medication use was reported by 46 % (*n* = 366) and, on average, the participants reported using acute medication 23.5 days/month. The frequency was lowest among the young and higher in older age groups (*r* = 0.18, *p* < 0.001). Among the youngest, the number of days/month with headache was greater than the number of days/month with medication use, whereas the opposite was true for those aged ≥30 years (Fig. 1). The mean number of days/month of medication use was higher among those who had only attended elementary school (24.4 days/month) than among those with high school education (23.0 days/month, *p* = 0.018).

More than half (53 %, *n* = 423) reported having used at least two different acute medications during the last 3 months, and the mean was 1.8 different acute medications (SD ± 0.9). Simple analgesics were most often the primarily used acute medication and opioids were more common among men than women (*p* = 0.018) (Table 2). The majority reported taking the primary acute medication orally (*n* = 754, 96 %), and use of prophylactic medication was reported by 11 % (*n* = 83) (Table 3). The proportion using prophylactics was smaller among those who only had elementary school education compared to those with university education (*p* = 0.021).

**Table 2:** Primary overused medication, reported by 785 individuals with medication-overuse headache (MOH)

Type of medication	Men		Women		Total		Age of user (years)		Frequency of use (days/month)^e^	
	*n*	%	*n*	%	*n*	%	Mean	SD	mean	SD
Triptan	10	5.5	55	9.0	65	8.3	49.9	12.4	21.7^b^	7.0
Ergotamine	2	1.1	5	0.8	7	0.9	59.3	15.0	21.1	9.1
Opioid	13	7.1^a^	19	3.2^a^	32	4.1	55.0	14.2	27.5^b,c,d^	4.9
Combination analgesic	48	26.2	125	20.8	173	22.0	51.7	13.0	23.9^c^	6.9
Simple analgesic	110	60.1	398	66.1	508	64.7	50.4	16.0	23.4^d^	6.7
Total	183	100	602	100	785	100	50.9	15.1	23.3	6.8

Values marked with the same letter are significantly different from each other (Pearson Chi square test and ANOVA with post hoc test Tukey’s HSD were used) (^a^ *p* = 0.018, ^b^ *p* = 0.0010, ^c^ *p* = 0.041, ^d^ *p* = 0.0063)

^e^Frequency of medication use was reported as the mean number of days/month over the last 3 months

**Table 3:** Medication use among individuals with medication-overuse headache (MOH), illustrated by the proportions using over-the-counter (OTC) acute medications and prophylactic medication

Parameter	Total (*n*)	Proportion only buying OTC (*n* = 785)		Proportion using prophylactics (*n* = 782)	
		*n*	%	*n*	%
Total	799	370	47.1	83	10.6
Sex					
Men	190	83	45.6	22	12.0
Women	609	287	47.6	61	10.2
Age (years)					
15–20	23	18	78.3^a,b,c^	0	0.0
21–29	46	32	71.1^f,g,h,i,j^	5	10.9
30–39	114	59	53.2^a,f,k,l^	14	12.5
40–49	192	92	48.4^b,g^	22	11.7
50–64	284	119	42.8^c,h^	28	10.1
65–74	89	33	37.1^d,i,k^	8	9.3
≥75	50	17	34.7^e,j,l^	5	10.2
Missing	1	0	–	1	–
Education					
Elementary school	296	114	39.4^m,n^	22	7.7^p^
High school	315	160	51.4^m^	35	11.2
University	182	93	52.0^n^	26	14.4^p^
Missing	6	3	–	0	–
Primary headache					
Migraine	394	167	43.2^o^	53	13.6^q^
Other headache	280	148	52.9^o^	18	6.4^q^
Missing	125	55	–	12	–

Values marked with the same letter are significantly different from each other (Pearson Chi square test was used): ^a^ *p* 0.027, ^b^ *p* = 0.0068, ^c^ *p* = 0.0010, ^d^ *p* < 0.001, ^e^ *p* < 0.001, ^f^ *p* = 0.039, ^g^ *p* = 0.0061; ^h^ *p* < 0.001, ^i^ *p* = <0.001, ^j^ *p* = <0.001, ^k^ *p* = 0.023, ^l^ *p* = 0.031, ^m^ *p* = 0.0032, ^n^ *p* = 0.0081, ^o^ *p* = 0.010, ^p^ *p* = 0.021, ^q^ *p* = 0.0032)

Almost half (47 %, *n* = 370) reported only using OTC medications (Table 3). This proportion was higher among the young than the old (*p* < 0.001, OR 0.98, 95 % CI 0.97–0.98) and lower among those who had only attended elementary school compared to those with high school education (*p* = 0.0032) or university education (*p* = 0.0081). Among those who used prescription medication, 82 % (*n* = 311) reported receiving all prescriptions from the same physician. This proportion did not differ according to the primary medication, e.g. between those using psychotropics (*n* = 64, 81 %) and those using other medications (*n* = 246, 82 %) (*p* = 0.78).

Thirty-two individuals reported using an opioid as the primary acute medication and 51 used a combination analgesic containing opioids. Thus, 10 % (*n* = 83) used a psychotropic medication as primary acute medication. The proportion was higher among men (16 %, *n* = 31) than women (8.5 %, *n* = 52) (*p* = 0.0022). Both the frequencies of headache and of medication use were higher among those using psychotropics (25.5 days/month, SD ± 5.9 and 27.1 days/month, SD ± 5.2, respectively) than among those using other medications (22.5 days/month, SD ± 6.2 and 23.1 days/month, SD ± 6.8) (*p* < 0.001 in both cases). Those using psychotropic medications were older (mean age 55 years, SD ± 14) than those using other medications (mean age 51 years, SD ± 15) (*p* = 0.011) and had made more visits to their physician (*p* = 0.0040) (Table 4).

**Table 4:** Health-care contacts among 799 individuals with medication-overuse headache

Parameter	Total (*n*)	Number of visits to the physician last year (*n* = 746)		Proportion who had seen a neurologist during last year (*n* = 746)		Proportion who had been informed about MOH (*n* = 785)	
		Mean	SD	*n*	%	*n*	%
Total	799	1.7	3.8	102	13.7	362	46.1
Sex							
Men	190	2.0	4.1	25	13.2	79	43.4
Women	609	1.6	3.6	77	12.6	283	46.9
Age (years)							
15–20	23	2.4	4.8	3	13.0	8	34.8
21–29	46	1.7	3.7	9	19.6	13	28.3
30–39	114	1.9	3.5	14	12.3	55	49.5
40–49	192	1.9	4.8	23	12.0	80	42.1
50–64	284	1.5	3.2	42	14.8	151	54.3
65–74	89	1.7	3.9	7	7.9	37	42.0
≥75	50	1.1	2.0	4	8.0	17	35.4
Missing	1	–	–	0	–	1	–
Educational level							
Elementary school	296	1.8	3.7	30	10.1^d^	131	45.5
High school	315	1.6	3.0	48	15.2^d^	143	45.8
University	182	1.7	5.1	23	12.6	88	49.2
Missing	6	–	–	1	–	0	–
Primary headache							
Migraine	394	2.0^a^	4.5	55	14.0	205	52.7^f^
Other headaches	280	1.3^a^	2.8	35	12.5	101	36.1^f^
Missing	125	–	–	12	–	56	–
Prescription status							
On prescription	415	2.6^b^	4.7	85	20.5^e^	228	55.6^f^
Always OTC	370	0.7^b^	1.9	16	4.3^e^	131	35.4^f^
Missing	14	–	–	1	–	3	–
Primary acute medication							
Psychotropic	83	3.2^c^	4.8	21	25.3	54	65.9^g^
Not psychotropic	716	1.5^c^	3.6	81	11.3	308	43.8^g^
Type of physician							
Neurologist	102	4.0	4.2	×	×	66	64.7
Other physician	241	3.1	3.2	×	×	132	55.5

*OTC* over the counter

Values marked with the same letter are significantly different from each other (*t* tests and Pearson Chi square tests were used): ^a^ *p* = 0.021, ^b^ *p* < 0.001, ^c^ *p* = 0.0040, ^d^ *p* = 0.046, ^e^ *p* < 0.001, ^f^ *p* < 0.001, ^g^ *p* < 0.001

### Health-care contacts

On average, the participants had made 1.7 visits to their physician due to headache during the last year (SD ± 3.8, range 0–52) (Table 4). In the same period, less than half (46 %, *n* = 343) had visited their physician at all and 14 % (*n* = 102) had seen a neurologist. The proportion was lower among those who had only attended elementary school than among those who had a high school education (*p* = 0.046). Less than half (46 %, *n* = 362) reported ever having received information about MOH from a physician (Table 4). This proportion was larger among those who used prescription medications compared to those who only used OTC medications (*p* < 0.001), and those who had received information had made more physician visits (2.3 visits) than those who had not been informed (1.1 visits) (*p* < 0.001).

### Sickness absence

The majority (*n* = 354, 79 %) of those in the working age group (18–64 years, *n* = 446) reported no headache-related sickness absence at all during the last 3 months (Table 5). Among the 92 who did, the mean was 14.7 days/month, being higher among men than women (*p* = 0.032). The proportion with headache-related sickness absence was higher in the youngest age group (18–20 years, 54 %) than in all other age groups and higher in the group who used psychotropic medications compared to those using other medications (*p* < 0.001) (Table 5).

**Table 5:** Headache-related sickness absence among those of working age (18–64 years) with MOH (*n* = 446)

Parameter	Total (*n*)	Proportion with sickness absence ≥1 day		Sickness absence among those with ≥1 day (days/month)	
		*n*	%	Mean	SD
Total	446	92	20.6	14.7	13.1
Sex					
Men	92	20	21.7	20.2^i^	13.0
Women	354	72	20.3	13.3^i^	12.8
Age (years)					
18–20	13	7	53.8^a,b,c,d^	9.9	9.8
21–29	41	7	17.1^a^	19.9	5.2
30–39	83	20	24.1^b^	11.9	13.4
40–49	145	34	23.4^c,e^	15.9	13.0
50–64	164	24	14.6^d,e^	15.0	13.7
Educational level					
Elementary school	123	23	18.7	18.4	12.8
High school	208	53	25.5^f^	14.6	13.3
University	113	16	14.2^f^	9.4	11.8
Missing	2	–	–	–	–
Primary headache					
Migraine	225	61	27.1^g^	13.9	13.0
Other headaches	153	22	14.4^g^	17.9	13.2
Missing	68	–	–	–	–
Primary acute medication					
Psychotropic	38	17	44.7^h^	19.0	13.8
Not psychotropic	408	75	18.4^h^	13.7	12.8

Values marked with the same letter are significantly different from each other (Pearson Chi squares test and* t* tests were used): (^a^ *p* = 0.0084, ^b^ *p* = 0.027, ^c^ *p* = 0.017, ^d^ *p* < 0.001, ^e^ *p* = 0.048, ^f^ *p* = 0.030, ^g^ *p* = 0.0033, ^h^ *p* < 0.001, ^i^ *p* = 0.032)

## Discussion

This is the first population-based study of medication use, health-care contacts and sickness absence among MOH-sufferers. We found that almost half only used OTC medications, that less than half had made a headache-related visit to their physician during the last year and that only 14 % had consulted a neurologist during the corresponding period. Those using psychotropic medications seemed to suffer from a greater disease burden than those using other medications. There were several important sociodemographic differences. Men with MOH reported a higher frequency of headache than women and older MOH-sufferers medicated more frequently than younger individuals. Both the use of medications and health-care differed in relation to educational level.

### Health-care contacts, medication use and sickness absence

We found that 44 % had made a headache-related visit to the physician during the last year. Previous population-based figures in MOH are lacking but the finding is in line with that found in a study of chronic daily headache (CDH), in which Scher et al. [19] reported a corresponding figure of 46 %. Only 14 % of our participants had consulted a neurologist during the last year and less than half could remember ever having been informed about MOH by a physician. This proportion was larger among those who used prescription medications than among those only using OTC medications, but then it is important to consider that almost half of the participants reported only using OTC medications. These findings suggest that many MOH-sufferers do not have regular contact with health-care providers. We find this remarkable considering the disease burden that is indicated by the reported frequencies of headache and medication use. A previous Swedish population-based study on migraine showed that 73 % had stopped seeing or had never seen a physician for their headaches [12]. A possible explanation for these low consultation rates may be found in a qualitative study of migraine and CDH by Peters et al. [20]. They reported that some patients had low expectations and that they questioned the physicians’ ability and interest to treat headaches to the extent that they chose not to consult for headaches [20]. The findings may also be a result of limited access to headache care. We believe that it is crucial to encourage increased contact between headache sufferers and health-care, and that in order to reach this patient group, preventive work should include other actors, such as pharmacies and other traders that sell OTC medications.

One in ten participants reported using a psychotropic medication as the primary acute medication. Colas et al. [4] found a corresponding figure of 12.5 % in their population-based study. In the present study we found several differences between those using psychotropics and those who did not, e.g. the frequencies of headache and of medication use, the proportion with headache-related sickness absence and the number of physician visits were all higher among the former. The higher consultation rate may partly be explained by the fact that no psychotropic medications are available without prescription in Sweden. The differences suggest that MOH-sufferers using psychotropic medications are more bothered by their disorder than those using other medications. Since this was a cross-sectional study, causality is unknown. The findings may, however, be interpreted as support for the suggestion that MOH-sufferers who overuse psychotropic substances should be regarded as a specific, more severe subgroup of MOH [6–8].

One-fifth of the participants reported headache-related sickness absence. This proportion could be regarded as small given that more than one-third reported having daily headaches. However, the finding may partly be explained by results reported by Ferrari et al. [21], who found that more than half of those with headache reported taking an analgesic and continuing working if the headache came during the working day. Among the 20 % who did report sickness absence in our study, the mean monthly frequency was as high as 15 days/person. Sickness absence thus seems to be skewed in the sense that only a small proportion reported headache-related sickness absence, but within this proportion the rate of sickness absence was high.

### Sociodemographic differences

Men with MOH reported a higher frequency of headache than women. To our knowledge, this difference has not been reported previously and contrasts with what is usually reported for the primary headaches [22]. Interestingly, despite their higher frequency of headache, men did not report using acute medications more often than women. Men also had a higher rate of headache-related sickness absence than women. Most other studies concerning sickness absence and headache have reported higher figures among women [23, 24]. However, the latter finding could be a reflection of the higher frequency of headache found among men with MOH in this study. Furthermore, overuse of psychotropics was more common among men than women and since sickness absence was associated with the use of psychotropics, this may have contributed to the sex-difference in sickness absence.

The frequency of medication use differed with age, being the lowest among the young, whereas the frequency of headache did not show the same age pattern. In fact, the frequency of headache was higher than the frequency of medication use among the youngest, whereas the opposite was seen in the older age groups. There was also an association between older age and a larger proportion using prescription medications. A similar relation was observed among migraineurs by Linet et al. [25], who found that the proportion using prescription medications was almost twice as high among young men aged 18–29 years than among boys aged 12–17 years old. Further, the headache-related sickness absence in our study was surprisingly high among the youngest. This group was small but the proportion reporting sickness absence was nevertheless significantly higher than in all other age groups. Since a high rate of sickness absence is a known risk factor for ending up more permanently outside the labour market with sickness benefit or social welfare [26], early identification of young individuals with MOH is important. A recent Danish study showed that medication use for headache follows a behavioural pattern that may track from adolescence into adulthood [27], thus further underlining the need for early identification and more research on the strategies used by young headache sufferers in order to manage headache.

Previous studies have shown that MOH is more prevalent among those with a low educational level [15, 17]. In the present study, several significant differences relating to educational level were detected, e.g. both the frequencies of headache and of medication use were higher among those who only had elementary school education than among the more highly educated. In a large prospective study, Hagen et al. [28] showed that low socioeconomic status was indeed a risk factor for frequent headache, but this has yet to be confirmed for MOH specifically. We also found that those with a lower educational level were less likely to use prophylactic medication or to have consulted a neurologist than those with a higher educational level. These findings suggest that the use of medications and health-care is unequal in relation to educational level among individuals with MOH in Sweden. Such differences are not in line with the Swedish health-care act, which states that health-care should be provided to everyone, on equal terms [29]. There is a need for longitudinal research in order to evaluate the consequences of these differences and to analyse whether they are the result of health-care actually being provided unequally or if help-seeking behaviours differ in relation to educational level. Similar differences were recently found in a Swedish study on epilepsy patients, in which socio-economic characteristics were important for access to neurologists and the prescriptions of individual antiepileptic medications [30]. The authors suggested differences in help-seeking behaviour as a possible explanation.

### Methodological considerations

A major methodological strength is the large sample size, which was based on the entire Swedish population, aged ≥15 years. The sample was somewhat skewed towards containing a larger proportion of women and elderly compared to the general population but is still considered representative of the Swedish population, aged ≥15 years in 2009. A thorough discussion of the representativeness of the sample has been published previously [17]. Though the overall study population is large, there are limited counts in some of the subgroups. Another potential limitation is that the interviewers were not headache specialists. However, in two previous studies comparing structured interviews conducted by lay interviewers with headache specialist ratings, the agreement between the two was validated [31, 32]. Both studies used the same diagnostic criteria for MOH as in the present study [2]. All data in this study are based on self-report and the risk of recall bias is thus a potential limitation. However, previous studies comparing the self-reported use of health-care resources and medications with registry data have shown high concordance between the two [33], even when patients were interviewed over the telephone [34].

## Conclusions

The results of this population-based study showed that many MOH-sufferers have limited contact with health-care institutions. In order to reach this patient group with preventive measures, we therefore recommend involving additional actors, such as pharmacies and other traders that sell OTC medications. Since we detected several differences suggesting that the use of medications and health-care among MOH-sufferers in Sweden is unequal with regard to educational level, particular effort should be directed towards those with a low educational level. Another group that warrants particular effort is MOH-sufferers using psychotropic medications. These individuals differed from others in several ways, suggesting that they suffer from a greater disease burden. Finally, young individuals with MOH differed from older individuals in the sense that they medicated less frequently and that they tended to use OTC medications rather than prescription medications. Many of these young individuals are most likely at the beginning of their disease career and more research on this group and their coping strategies could shed valuable light on the development from primary headache to MOH.
